# Diagnostic and Therapeutic Challenges Related to HER2 Heterogeneity in Gastric Cancer: Bridging Molecular Pathology and Clinical Decision-Making

**DOI:** 10.3390/ijms27031542

**Published:** 2026-02-04

**Authors:** Nelia Marina Rosanu, Lorenzo Gervaso, Renato Lobrano, Alessandro Vanoli, Chiara Alessandra Cella, Nicola Fusco, Nicola Fazio

**Affiliations:** 1 European Institute of Oncology, IEO IRCCS, 20141 Milan, Italy; 2 Department of Oncology, Elias Emergency University Hospital, 011461 Bucharest, Romania; 3 Division of Gastrointestinal Medical Oncology and Neuroendocrine Tumors, European Institute of Oncology, IEO IRCCS, 20141 Milan, Italy; 4 Division of Pathology, European Institute of Oncology, IEO IRCCS, 20141 Milan, Italy; 5 Department of Molecular Medicine, University of Pavia, 27100 Pavia, Italy; 6 Unit of Anatomic Pathology, IRCCS San Matteo Hospital Foundation, 27100 Pavia, Italy; 7 Department of Oncology and Hemato-Oncology, University of Milan, 20122 Milan, Italy

**Keywords:** HER2, gastric cancer, heterogeneity, HER2-low, molecular pathology, retesting, precision oncology

## Abstract

HER2 testing represents a cornerstone of the treatment algorithm in advanced gastric and gastroesophageal junction adenocarcinoma (GC), yet its evaluation remains complex due to tumor heterogeneity and methodological variability. Unlike breast cancer, HER2 expression in GC is often incomplete and heterogeneous, resulting in discordant results between biopsies, resections, and metastatic sites. Both spatial and temporal HER2 heterogeneity are key determinants of testing reproducibility, diagnostic accuracy, and treatment selection and response in GC. Optimizing sampling through multiple, well-targeted biopsies, standardizing IHC/ISH protocols, and reassessing HER2 status at progression may be crucial steps to ensure diagnostic accuracy. The recognition of HER2-low disease introduces a new pathological and clinical subgroup of GC with potential sensitivity to antibody–drug conjugates, while emerging techniques such as circulating tumor DNA analysis are increasingly applied to detect HER2 amplification and co-existing genetic alterations. Integrating molecular tools and standardized reassessment strategies can enhance HER2 testing reliability and enable more precise treatment strategies, with the potential to minimize HER2 resistance mechanisms. This review provides a practice-oriented guide on the interpretation and optimization of HER2 testing in gastric cancer, while providing insight into the underlying molecular mechanisms driving heterogeneity and resistance.

## 1. Introduction

### 1.1. HER2 Testing in Gastric Cancer

The evaluation of human epidermal growth factor receptor 2 (HER2), also known as Erb-b2 receptor tyrosine kinase 2 (ERBB2), status in gastric and gastroesophageal junction adenocarcinoma, hereinafter referred to as gastric cancer (GC), represents a cornerstone of personalized management [1]. Determining HER2 status at diagnosis is essential, as it directly guides eligibility for targeted therapies in advanced disease and influences treatment strategies throughout the disease course [2]. Besides its prognostic value, HER2 primarily serves as a predictive biomarker, making the accuracy and reproducibility of testing results critical for its applicability in clinics [2,3]. An incorrect classification may either exclude patients from effective therapies or expose them to treatments unlikely to provide benefit [1]. The marked tumor heterogeneity and modulation of HER2 expression, mirroring dynamic molecular reprogramming across genomic, epigenetic, and signaling layers, further underscore the importance of reassessment in selected scenarios, such as disease progression or recurrence [4,5,6]. Accurate HER2 evaluation remains crucial to treatment decision-making and personalized oncology [6]. The main focus of this review is to summarize current knowledge on HER2 biology, diagnostic challenges, and emerging translational strategies in GC.

### 1.2. Literature Search Strategy

As scientific background, we performed a selective literature search in PubMed and Cochrane databases up to August 2025, using the keywords “gastric cancer,” “HER2,” “HER2 testing,” “positivity loss,” “positivity gain,” “retest,” “rebiopsy,” “conversion,” “reassessment,” “trastuzumab,” “primary lesion biopsy,” “metastatic lesion biopsy,” and “HER2 resistance.” Landmark clinical trials, international guidelines, and key pathological and translational studies were prioritized, while additional references were identified through the bibliographies of relevant publications. Only English-language, peer-reviewed literature was considered. Evidence was synthesized with expert interpretation to highlight clinically relevant challenges in HER2 testing and their implications for therapeutic decision-making. Given the narrative nature of this review, no formal quality assessment of studies has been performed.

### 1.3. Pathological Standards for HER2 Testing in Gastric Cancer: State of the Art

The assessment of HER2 status in GC is currently performed by immunohistochemistry (IHC) ± in situ hybridization (ISH) techniques, according to the College of American Pathologists (CAP) [7]. To date, genomic profiling alone is insufficient to replace conventional HER2 assessment [8]. According to CAP guidelines, HER2 testing in GC requires adequately fixed and representative tissue, focusing on invasive areas and using validated IHC/ISH protocols [8,9]. HER2 evaluation should focus exclusively on the invasive component, selecting blocks with abundant viable tumor, preferably of lower histologic grade. Scoring follows the Rüschoff and Hofmann system, both for IHC and ISH, developed specifically for GC. In this system, HER2 positivity (IHC 3+) is defined by strong membranous staining intensity, with either complete or incomplete (basolateral or lateral) staining patterns, when present in ≥10% of tumor cells in resection specimens or in clusters of ≥5 cohesive tumor cells in biopsies [10]. Cases with weak-to-moderate membranous staining (IHC 2+) are considered equivocal and require confirmation by ISH, where amplification is defined as a HER2/chromosome 17 centromere enumeration probe (CEP17) ratio ≥ 2.0. This approach reflects the basolateral and heterogeneous HER2 staining pattern typical of GC, which differs from the uniform circumferential membranous pattern observed in breast cancer [1,11]. Despite this difference in staining distribution, the assessment of staining intensity follows the same grading system in both settings, classified as absent, weak, moderate, and intense [12].

Guidelines recommend an initial IHC evaluation, with scores ranging from 0 to 3+. Results are interpreted as follows: 0–1+ (negative), 2+ (equivocal, requiring ISH confirmation), and 3+ (positive) [13]. Based on data from the TOGA trial, this method yielded a 93.5% concordance between IHC and ISH, later confirmed by other studies reporting rates between 87% and 98% [2].

Several pre-analytical factors can influence HER2 assessment, including formalin fixation time and technique, clone selection (e.g., 4B5 vs. A0485), and interobserver variability, all of which affect the reproducibility of the HER2 scoring, particularly for the HER2-low classification [14]. To ensure accurate HER2 evaluation, biopsy or resection specimens should be rapidly placed in fixative, ideally within 1 h of collection, and fixed in 10% neutral buffered formalin for 6–72 h. Subsequent tissue processing and HER2 testing should follow analytically validated laboratory protocols, as suboptimal specimen handling or fixation may compromise assay reliability and result interpretation [8]. HER2-low status in gastric cancer introduces additional challenges in reproducibility and interobserver concordance, particularly in biopsy specimens, and may highlight the need for further refinement of pre-analytical protocols as therapies targeting HER2-low tumors continue to evolve.

### 1.4. HER2 Signaling and Resistance Mechanisms

Comprehensive molecular profiling from The Cancer Genome Atlas (TCGA) classified GC into four subtypes: EBV (Epstein–Barr Virus)-positive, microsatellite instability (MSI), genomically stable, and chromosomal instability (CIN) [15]. The ERBB receptors belong to the receptor tyrosine kinase family and regulate key cellular processes, including growth, survival, and repair. In gastric cancer, *ERBB2* amplification occurs predominantly in CIN tumors and leads to HER2 overexpression with sustained oncogenic signaling [7,15]. HER2 frequently heterodimerizes with other ERBB receptors, particularly HER3 and EGFR, thereby activating key intracellular pathways such as PI3K/AKT/mTOR and RAS/RAF/MEK/ERK, which promote tumor proliferation and resistance to apoptosis. HER2–HER3 complexes are potent activators of PI3K–AKT signaling through multiple PI3K-binding motifs on HER3, whereas HER2–EGFR heterodimers can strongly engage MAPK signaling [6,7,9].

**Figure 1:**
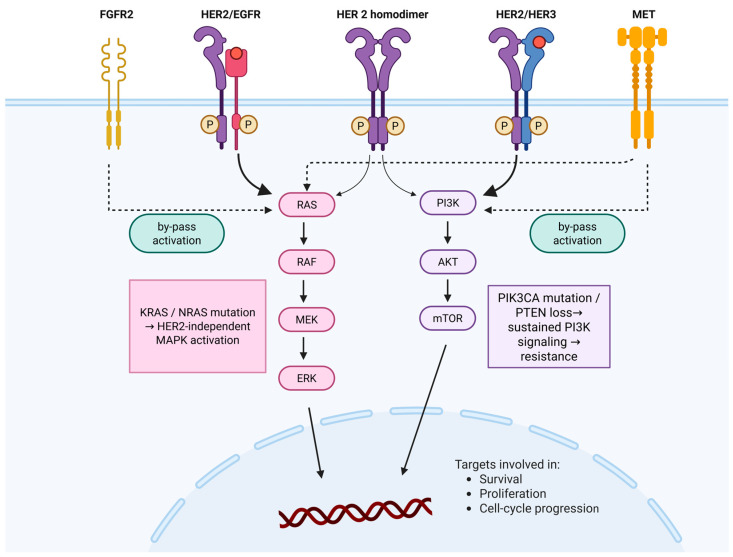
Molecular pathways of HER2 activation and resistance. Solid arrows indicate direct downstream HER2-driven signaling, whereas dashed arrows represent dominant bypass or co-activation signaling pathways contributing to resistance. (created with BioRender.com).

Recent genomic studies further show that HER2-positive tumors are molecularly heterogeneous, and may harbor co-alterations that modulate treatment response and contribute to resistance [5,7,15]. These include the activation of parallel receptor tyrosine kinases such as FGFR2 or MET, which can bypass HER2 inhibition, as well as downstream alterations, including PIK3CA mutations, KRAS or NRAS mutations, and PTEN loss, that may sustain MAPK or PI3K–AKT signaling independently of HER2, collectively contributing to primary or acquired resistance to anti-HER2 therapies [5,6,7] (Figure 1). From a translational perspective, the multi-level organization of HER2 signaling provides a framework for pathway interference at distinct signaling nodes, supporting the interpretation of heterogeneous therapeutic responses.

## 2. Complexities in HER2 Assessment

### 2.1. Spatial Heterogeneity

Two main types of spatial heterogeneity in HER2 expression are recognized: intratumoral (intralesional)—variability within a single lesion, either the primary tumor (T–T) or within a distant metastatic/regional nodal lesion (M–M, N–N)—and interlesional, differences between distinct lesions, such as primary tumor and distant metastasis or regional nodal metastasis (T–M, T–N) or among different metastatic sites (M–M, N–M) [16]. The mechanisms underlying HER2 heterogeneity remain incompletely understood, but likely reflect the evolution and selection of molecularly distinct subclones during tumor progression (for example, the emergence of HER2-amplified clones within an otherwise HER2-negative tumor) [17,18].

#### 2.1.1. Intralesional and Interlesional HER2 Heterogeneity: How Do They Influence Clinical Management in Gastric Cancer?

**Figure 2:**
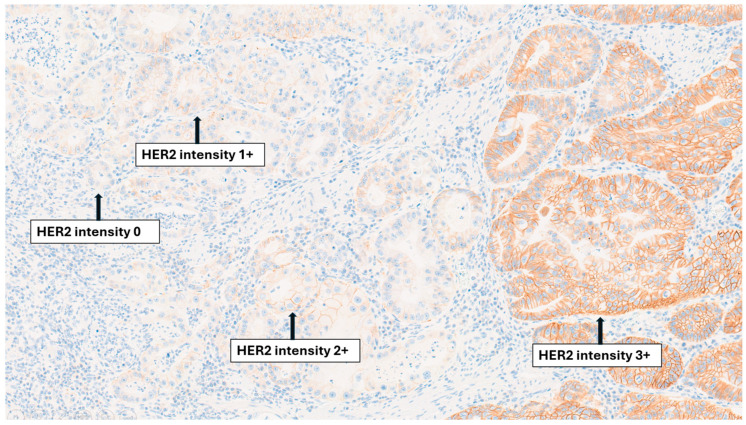
HER2 heterogeneity in gastric cancer (12× magnification).

Intratumoral spatial heterogeneity has been reported in 2.5–14% of all GCs and up to 23–74% of HER2-IHC positive or HER2-amplified GCs. Figure 2 illustrates variable HER2 staining intensities within a single GC, highlighting the coexistence of 0, 1+, 2+, and 3+ areas. Definitions of HER2 heterogeneity vary considerably across studies, from <30% HER2-positive cells [1] to 5–50% or 10–50% (IHC 2+/3+) or <50% amplified spots by ISH [19,20,21,22]. Intratumoral heterogeneity, which is more frequently observed in tumors with an intermediate IHC score (2+) and in diffuse or mixed Lauren subtypes, plays a major role in the discordance of HER2 status between biopsy and surgical specimens [19,22]. Because endoscopic biopsies may sample areas with limited or absent HER2 expression, and surgical specimens often reveal focal amplification, marked variability has been reported across studies, ranging from 28% in the cohort by Wang et al. to 54% in that of Ahn et al. [21,23]. Additionally, Fusco et al. reported that high-grade dysplasia showed concordance with invasive carcinoma in <50% of HER2 2+/3+ cases, emphasizing the importance of assessing HER2 expression in clearly invasive tumor components, which may be underrepresented in small endoscopic biopsies of primary tumor [24]. Therefore, endoscopic sampling may underestimate HER2 heterogeneity, limiting the accuracy of diagnostic biopsies. Interlesional heterogeneity, though less studied, occurs in 1–14% of cases [22]. It may result from the heterogeneity of the primary tumor or genomic differences between different metastatic sites [25,26,27]. Both the gain and loss of HER2 expression between the primary tumor and metastatic site have been described, as reported in recent multicentric studies. In the GASTHER1 study, “rescued” HER2 positivity was identified in 8.7% of patients through repeat endoscopic biopsies, and in 5.7% through the assessment of recurrent/metastatic lesions [28]. In another series comparing primary tumors and peritoneal metastases, discordance was 2.7% when categorized as HER2-positive/-negative, but 27% when graded as 0/low/high, underscoring the importance of reassessing metastases [29].

#### 2.1.2. Sampling Adequacy: How Can We Optimize HER2 Testing Accuracy in Gastric Cancer?

Spatial heterogeneity has direct clinical implications, as it has been reported to predict poorer response and survival in HER2-positive GC patients treated with trastuzumab [30,31]. Although its predictive value may be mitigated by the bystander effect of antibody–drug conjugates (ADCs) such as trastuzumab-deruxtecan (T-DXd), accurate assessment remains critical [32].

To improve diagnostic reliability, practical recommendations for endoscopists and pathologists include:

- Obtain >6 tumor-containing biopsies, preferentially from central viable tumor regions, while avoiding ulcerated or necrotic areas, which are more likely to be HER2-negative. [8,33,34,35].
- Re-perform biopsy of the primary lesion when initial HER2 status is negative or low.
- Quantitative reporting (exploratory): the H-score, which integrates staining intensity and proportion, may correlate with improved survival at higher values (>210), but is not recommended for routine HER2 assessment [36].
- Test multiple (≥2) formalin-fixed paraffin-embedded (FFPE) blocks in surgical specimens to increase sensitivity [19,37].
- Select representative blocks encompassing morphologic heterogeneity, particularly intestinal or differentiated areas, which are more likely to express HER2 [38].
- Whenever feasible, perform a biopsy of both primary and metastatic sites to capture heterogeneity.

The multicontinental LEGACY project validated HER2 status using standardized IHC protocols with NGS-based confirmation of ERBB2 amplification, demonstrating full concordance across laboratories and emphasizing the importance of standardized methodology [39].

### 2.2. Temporal Heterogeneity and Resistance Mechanisms

#### 2.2.1. Understanding the Impact on Treatment Decisions: Should We Retest HER2?

Temporal heterogeneity in HER2 expression refers to changes in HER2 status over time in the same patient. Similarly to spatial heterogeneity, it plays an important role in the development of treatment resistance and disease progression [16]. Several studies have reported discrepancies in HER2 status between sequential samples, such as initial biopsies and surgical specimens or between primary and metastatic sites [19,40]. These differences may reflect dynamic tumor evolution, clonal selection under treatment pressure, or technical factors related to tissue sampling and interpretation [41].

Two clinical situations illustrate this phenomenon: HER2-positive tumors that lose HER2 expression after trastuzumab-based therapy, and initially HER2-negative tumors that acquire HER2 amplification at recurrence, becoming potential candidates for targeted therapy in later treatment lines [42,43,44].

Overall, temporal HER2 heterogeneity may result from intrinsic mechanisms, reflecting the genetic instability and natural evolution of GC, or from adaptive mechanisms driven by exposure to therapy. The main processes involved include tumor clonality, epigenetic changes, and cellular plasticity (Table A1).

- Tumor clonality: GC harbors genetically distinct subclones that evolve under treatment. ERBB2-amplified clones may coexist or be replaced by populations driven by other RTKs (e.g., EGFR, MET, and FGFR2) or downstream activators (e.g., PIK3CA mutations, PTEN loss), sustaining signaling despite HER2 blockade [16,45,46].
- Extrachromosomal DNA (ecDNA): ecDNA elements carrying RTK genes (ERBB2, FGFR2, and MYC) can dynamically expand or contract under drug pressure, promoting resistance and rapid tumor adaptation. In GC models, ecDNA remodeling involving FGFR2 and MYC generated reversible resistant subclones, escaping HER2-targeted therapy [45,47].
- Epigenetic regulation: DNA methylation, histone modification, and non-coding RNAs (miRNAs/lncRNAs) can modulate ERBB2 transcription independently of copy number, explaining IHC variability despite stable ISH. These changes are potentially reversible and influenced by treatment pressure, inflammation, or microenvironmental stress [48,49].
- Tumor cell plasticity: Epithelial-to-mesenchymal transition (EMT), during which epithelial cancer cells lose adhesion and polarity and acquire a more mobile, mesenchymal-like phenotype, can reduce or abolish HER2 membrane expression, contributing to both diagnostic variability and therapeutic escape [50,51].

In addition, technical and pre-analytical factors related to tissue sampling and handling, including the selection of non-representative tumor areas, delayed or inadequate fixation, and suboptimal antibody selection, may confound the interpretation of apparent temporal changes in HER2 status, potentially leading to false-negative results, particularly in heterogeneous or HER2-low tumors [23,52,53].

While HER2 retesting is valuable to detect expression loss or gain, it does not capture all resistance mechanisms. Thus, re-evaluation should ideally be integrated with broader molecular profiling or circulating tumor DNA (ctDNA) analysis to comprehensively define the underlying biology.

#### 2.2.2. Timing, Technique, and Clinical Impact of HER2 Retesting

Recent trials, such as DESTINY-Gastric02, have confirmed the efficacy of T-DXd as a second-line therapy for HER2-positive GC, changing the treatment algorithm—also for subsequent lines [54,55]. Although initially HER2 confirmation was required for trial enrollment, a systematic rebiopsy after progression was not mandatory, and current clinical practice similarly does not require repeat testing before T-DXd if prior HER2 positivity was well established. Conversely, real-world data from GASTHER1 and GASTHER2 highlighted the occurrence of HER2 status conversion during disease evolution [28,44]. These findings underscore the clinical relevance of reassessment, particularly after progression or prolonged anti-HER2 therapy.

From a clinical standpoint, HER2 retesting may be indicated in three main scenarios:

- After tumor progression on first-line trastuzumab-based therapy, to verify persistence of HER2 positivity;
- After long-term anti-HER2 exposure, to identify potential HER2 loss;
- In some cases of HER2-negative tumors at recurrence, since conversion to HER2-positive occurs in ≈4–9% of cases according to GASTHER1/2 data.

Among these three situations, HER2 status conversion to positivity has the most impactful clinical implications: current treatment algorithms link anti-HER2 therapy to HER2 status at the time of treatment decision [13]. Because trastuzumab is approved only as the first line and T-DXd only after prior trastuzumab exposure, patients who become HER2-positive only at recurrence may miss the eligibility for first-line trastuzumab and may access T-DXd mainly through clinical trials or individualized off-label use. A reliable stratification of patients at risk of HER2 conversion is currently lacking and remains crucial to optimize treatment allocation and prevent missed therapeutic opportunities.

**Figure 3:**
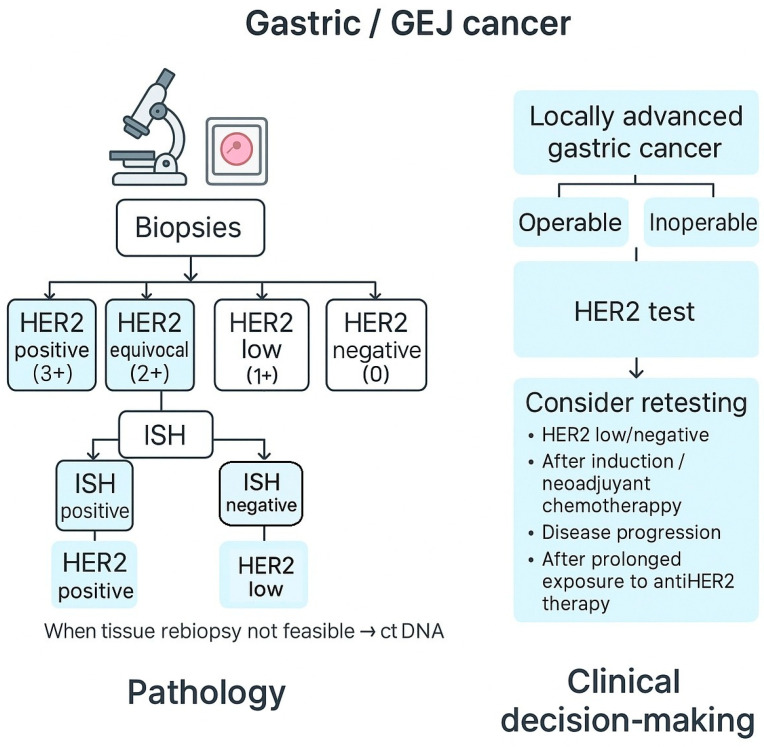
Workflow for HER2 testing and retesting in gastric and gastroesophageal junction cancer.

Testing should follow validated protocols identical to baseline evaluation and, whenever possible, be performed on newly obtained tissue from metastatic or recurrent sites [13,56,57]. The use of multiple tissue blocks and review by expert pathologists increases accuracy and minimizes sampling bias. Figure 3 summarizes the integrated pathology–clinical workflow for HER2 assessment. Recognizing the dynamic behavior of HER2 in GC calls for a more adaptive diagnostic strategy, balancing timing, technique, and clinical context.

### 2.3. HER2-Low

#### 2.3.1. HER2-Low Status: Histopathological Criteria and Interpretation

HER2-low GC is defined as IHC 1+, corresponding to faint or barely perceptible membranous staining, or as IHC 2+ without gene amplification (ISH-). Although a universal definition is lacking, these thresholds adapt gastric-specific scoring systems [1,11].

Distinguishing IHC 0 from 1+ remains challenging, particularly in poorly differentiated, mucinous, or heterogeneous tumors [11,22]. Pre-analytical factors such as fixation, antigen retrieval, and tissue preservation may further impact staining and classification [8]. Comprehensive HER2 reporting across the full 0–3+ spectrum is essential as therapeutic implications evolve.

#### 2.3.2. Therapeutic Implications

HER2-low GC may show intermediate molecular characteristics, including PIK3CA or KRAS mutations and the activation of PI3K/AKT or TGF-β signaling pathways. Some reports also suggest enhanced immune infiltration or programmed death-ligand 1 (PD-L1) expression in subsets of GCs lacking HER2 amplification [58].

While conventional anti-HER2 antibodies show limited efficacy in this setting, novel ADCs such as T-DXd have demonstrated activity through a bystander cytotoxic effect, even in tumors with heterogeneous or low HER2 expression, enabling the elimination of neighboring HER2-negative cells [58]. The drug’s efficacy in HER2-low tumors is attributed to its high drug-to-antibody ratio and membrane-permeable payload, enabling cytotoxic activity even in tumors with low or heterogeneous HER2 expression [3,46].

In DESTINY-Gastric02, clinical benefit from T-DXd was mainly observed in HER2-positive disease, although limited activity was noted in a small subset of HER2-low cases [55]. The efficacy of T-DXd in GC with low HER2 expression was also reported in a phase II study enrolling patients pretreated with ≥2 chemotherapy lines but anti-HER2-therapy naïve [46,59].

In current practice, HER2-low status is not yet an established therapeutic biomarker in GC, but consistent scoring and documentation may facilitate future trial eligibility.

### 2.4. HER2 in Locally Advanced Disease: Current Challenges and Emerging Clinical Evidence

In locally advanced resectable GC, HER2 testing at diagnosis contributes to tumor characterization and clinical trial selection, but its predictive role for perioperative therapy remains uncertain [13,56,60]. HER2 positivity in locally advanced GC seems to be lower than rates reported in pivotal clinical trials for advanced disease [2,61], ranging around 6% in retrospective studies [62,63]. Regarding the role of anti-HER2 agents in this setting, the PETRARCA and INNOVATION trials explored the addition of trastuzumab ± pertuzumab to perioperative chemotherapy, showing higher pathological response rates without an established definitive survival benefit [64,65]. Consequently, anti-HER2 agents are not standard in curative-intent protocols. Currently, the PHERFLOT/IKF-053 trial showed promising results with the addition of pembrolizumab and trastuzumab to preoperative FLOT [66].

Endoscopic biopsies obtained before neoadjuvant chemotherapy (NACT) often provide limited and fragmented tissue, which may not adequately represent the heterogeneous distribution of HER2 expression [37,38]. Because the treatment-induced modulation of HER2 expression may occur after NACT, the reassessment of the surgical specimen can provide additional biological information, though it is not routinely required [42,43]. For patients with locally advanced but unresectable HER2-positive GC, management is generally consistent with the management of metastatic disease. In this setting, trastuzumab plus platinum-based chemotherapy represents the standard first-line regimen [2]. In patients who may be eligible for downstaging, HER2 reassessment after induction therapy may support inclusion in clinical trials exploring perioperative anti-HER2 strategies.

### 2.5. Future Directions

Liquid biopsy using ctDNA sequencing is a fast-growing strategy for HER2 evaluation, particularly when tumor tissue collection is unfeasible. By capturing tumor-derived genomic alterations in plasma, ctDNA analysis may help overcome spatial heterogeneity across metastatic sites. Preliminary studies show 70–90% concordance with tissue HER2 assessment, though sensitivity varies with tumor burden and site [67,68]. Ongoing clinical trials are incorporating ctDNA analyses to refine HER2 assessment, identify resistance mechanisms, and guide treatment decisions, supporting its progressive inclusion in diagnostic workflows [3,69]. Beyond static testing, real-time ctDNA monitoring could enable the dynamic assessment of *ERBB2* amplification and emerging resistance alterations, providing a non-invasive strategy for longitudinal tracking of disease evolution [70]. In low- and middle-income countries, HER2 molecular testing is often limited by the restricted availability of validated IHC platforms, dual-probe ISH assays, and next-generation sequencing facilities, resulting in inconsistent diagnostic accuracy and under-identification of eligible patients for targeted therapies [39].

In this context, digital pathology and artificial intelligence (AI) offer complementary solutions to enhance diagnostic standardization and accessibility. Digital slide analysis enables centralized review, reduces interobserver variability, and improves the reproducibility of HER2 scoring [24,71]. Moreover, recent AI-driven convolutional neural network models have achieved accuracies exceeding 95% in both GC detection and HER2 evaluation [72]. These technologies are particularly valuable amid the growing diagnostic workload and shortage of specialized pathologists, offering a scalable, high-quality approach to HER2 assessment even in resource-limited settings [73].

## 3. Conclusions

HER2 testing stands at the intersection of pathology, molecular biology, and clinical oncology. Its reliability depends on both technical precision and a deep understanding of tumor heterogeneity. Variability in fixation, antibody selection, and interpretation can still lead to discordant results, emphasizing the importance of standardized, gastric-specific protocols and rigorous quality control programs. Clinically, HER2 remains a key predictive biomarker that guides the use of targeted therapies in advanced disease and, potentially, in early stages. However, its dynamic behavior, shaped by spatial and temporal heterogeneity, could require adaptive reassessment to ensure that treatment decisions reflect current tumor biology.

Looking ahead, integrating HER2 evaluation within molecular and digital frameworks may positively impact disease management. Real-time monitoring through ctDNA and multi-analyte liquid biopsy could allow for the dynamic assessment of *ERBB2* amplification and emerging resistance. Meanwhile, AI-assisted pathology offers the potential to enhance diagnostic reproducibility and harmonize HER2 quantification across institutions. By bridging molecular diagnostics with clinical decision-making, HER2 testing would shift from a static baseline classifier to a dynamic instrument for precision oncology in GC.

## Data Availability

No new data were created or analyzed in this study. Data sharing is not applicable to this article.

## Abbreviations

The following abbreviations are used in this manuscript:

GC	gastric and gastroesophageal junction adenocarcinoma
HER2	human epidermal growth factor receptor 2
ERBB2	Erb-b2 receptor tyrosine kinase 2
ERBB3/HER3	Erb-B3 receptor tyrosine kinase 3
IHC	Immunohistochemistry
ISH	in situ hybridization
CAP	College of American Pathologists
CEP17	chromosome 17 centromere enumeration probe
EGFR	epidermal growth factor receptor
MET	mesenchymal–epithelial transition factor
FGFR2	fibroblast growth factor receptor 2
PI3K	phosphoinositide 3-kinase
AKT	protein kinase B
mTOR	mechanistic target of rapamycin
MAPK	mitogen-activated protein kinase
RAF	rapidly accelerated fibrosarcoma kinase
MEK	mitogen-activated protein kinase kinase
ERK	extracellular signal-regulated kinase
RTK	receptor tyrosine kinase
PTEN	phosphatase and tensin homolog deleted on chromosome 10
PIK3CA	phosphatidylinositol-4,5-bisphosphate 3-kinase catalytic subunit alpha
RAS	rat sarcoma small GTPase
KRAS	Kirsten rat sarcoma viral oncogene homolog
NRAS	neuroblastoma RAS viral oncogene homolog
JAK	Janus kinase
STAT3	signal transducer and activator of transcription 3
EMT	epithelial-to-mesenchymal transition
TME	tumor microenvironment
TCGA	The Cancer Genome Atlas
MSI	microsatellite instability
CIN	chromosomal instability
ADC	antibody–drug conjugate
T-Dxd	trastuzumab-deruxtecan
FFPE	formalin-fixed paraffin-embedded
ecDNA	extrachromosomal DNA
miRNAs	microRNAs
lncRNAs	long non-coding RNAs
ctDNA	circulating tumor DNA
IL-6	Interleukin-6
TP53	tumor protein 53
CCNE1 PD-L1	Cyclin E1 programmed death-ligand 1
NACT	neoadjuvant chemotherapy
AI	artificial intelligence

## Appendix A

**Table A1:** Molecular mechanisms influencing HER2 expression.

Pathway	Biological Effect	Clinical Impact	Evidence	Target
**Receptor-level alterations**				
ERBB2 (HER2) amplification/overexpression	Constitutive receptor activation → persistent downstream signaling	Defines HER2-positive subtype; predicts benefit from trastuzumab and ADCs	A	✓
ERBB3 (HER3) activation/dimerization	Amplifies PI3K/AKT signaling even under HER2 blockade	Potential target for dual HER2/HER3 blockade	B	☆
EGFR or MET co-amplification	Parallel RTK signaling bypasses HER2 inhibition	Mechanism of acquired resistance; supports combination strategies	B	☆
FGFR2 amplification	Alternate RTK activation (escape from HER2 dependence)	May mediate anti-HER2 resistance; may benefit from FGFR inhibitors	B	☆
**Downstream signaling alterations**				
PIK3CA mutation/PTEN loss	PI3K/AKT/mTOR activation independent of HER2	Reduced trastuzumab sensitivity; PI3K/mTOR inhibitors investigational	B	☆
KRAS/NRAS mutations	Constitutive MAPK signaling independent of HER2	May confer primary resistance to HER2 blockade	B	•
**Epigenetic and transcriptional regulation**				
ERBB2 promoter methylation, histone deacetylation, miRNAs, lncRNAs	Downregulates HER2 transcription without copy-number change; post-transcriptional modulation of HER2	Explains temporal HER2 loss; reversible with epigenetic drugs; may serve as biomarkers of HER2 fluctuation or treatment resistance	C	☆
**Phenotypic and micro-environmental adaptation**				
Epithelial–mesenchymal transition (EMT)	Loss of cell adhesion and membrane HER2 expression	Decreased ADC uptake; associated with invasive phenotype	C	•
IL-6/JAK/STAT3 activation (inflammatory TME)	Cytokine-driven survival signaling with HER2 expression modulation	Promotes trastuzumab resistance; potential for JAK–STAT blockade	C	☆
**Genomic structural plasticity**				
Extrachromosomal DNA, chromosomal instability (*TP53*, *CCNE1* co-alterations)	Rapid oncogene (de-)amplification under therapy; increased intratumoral heterogeneity and clonal evolution	Potential dynamic resistance mechanism; rationale for repeat HER2 testing and multimarker profiling	A	•

**Legend**: ✓—actionable; ☆—investigational; •—not directly targetable. **Evidence**: A—proven in clinical trials or large translational cohorts; B—supported by preclinical or translational studies; C—emerging hypothesis or early evidence.
